# Assessing Well-Being Among Aging Women: Observations From the Women's Health Initiative

**DOI:** 10.1093/geroni/igab046.1818

**Published:** 2021-12-17

**Authors:** Kenneth Pike, Eileen Rillamas-Sun, Barbara Cochrane, Nancy Woods

**Affiliations:** 1 University of Washington, Seattle, Washington, United States; 2 Fred Hutchinson Cancer Research Center, Seattle, Washington, United States

## Abstract

Our aim was to develop a profile of well-being preserving the ability to estimate differential effects of both hedonic and eudaemonic dimensions of well-being on health outcomes. Numerous indicators of well-being from over 80,000 aging women included hedonic (enjoyment of life, happiness, satisfaction with life, quality of life) and eudaemonic (personal growth, purpose in life, environmental mastery, control, self mastery) dimensions. Using latent class analysis, we identified groups of women with distinct profiles of well-being. A four-class solution had both good statistical fit and made conceptual sense. Class 1 (n=9,146, 11%) had the lowest scores on hedonic and eudaemonic indicators, while Class 4 (n=38,246 47%) had the highest levels of all well-being indicators. Class 2 (n=7,106, 9%) had higher hedonic and lower eudaemonic scores and Class 3 (n=26,650, 33%) had lower hedonic and higher eudaemonic scores. These classes form a well-being profile useful for estimating differential effects on health outcomes.

